# Cytogenetic analysis of B chromosomes in one population of the fish *Moenkhausia sanctaefilomenae* (Steindachner, 1907) (Teleostei, Characiformes)

**DOI:** 10.3897/CompCytogen.v6i2.1769

**Published:** 2012-04-09

**Authors:** Diogo Teruo Hashimoto Tatiana Aparecida Voltolin, Ana Danyelle Noitel Valim de Arruda Paes, Fausto Foresti, Jehud Bortolozzi, Fábio Porto-Foresti

**Affiliations:** 1 Departamento de Ciências Biológicas, Faculdade de Ciências, Universidade Estadual Paulista (UNESP), Av. Eng. Luiz E. C. Coube, 17033-360, Bauru, SP, Brazil; 2 Departamento de Morfologia, Instituto de Biociências, Universidade Estadual Paulista (UNESP), Dist. Rubião Júnior, 18618-000, Botucatu, SP, Brazil

**Keywords:** fish cytogenetic, NOR expression, supernumerary chromosomes, mitotic instability

## Abstract

The aim of this study was to characterize cytogenetically one population of the fish *Moenkhausia sanctaefilomenae* (Steindachner, 1907), with emphasis on the analysis of B chromosomes. The nucleolar activity in the B microchromosomes was characterized, and an analysis of mitotic instability of these microchromosomes was accomplished. The results showed a diploid chromosome number of 50 chromosomes. In all individuals, we observed the presence of B microchromosomes with intra- and inter-individual variability. The analysis of the nucleolus organizing regions (NORs) by silver nitrate staining demonstrated multiple NORs. We observed active sites of ribosomal DNA in the B microchromosomes, with a frequency of 20% in the analyzed cells, which shows gene activity in these chromosomal elements. The analysis of constitutive heterochromatin patterns showed that the B microchromosomes are heterochromatic or euchromatic, which demonstrates differentiation of DNA composition between these genomic elements. The calculation of the mitotic instability index implied that B chromosomes in this species might be in a final stage of instability.

## Introduction

*Moenkhausia* Eigenmann, 1903 is considered as *incertae sedis* in Characidae and contains 65 valid species widely distributed in the Neotropical river basins (Lima et al. 2003). Although the genus *Moenkhausia* cannot be characterized as monophyletic, a group consisted of *Moenkhausia oligolepis* (Günther, 1864), *Moenkhausia sanctaefilomenae* (Steindachner, 1907), *Moenkhausia cotinho* Eigenmann, 1908, and *Moenkhausia pyrophthalma* Costa, 1994 shares a very similar color pattern (Costa 1994). *Moenkhausia* systematic is very complex and nowadays several studies have shown that it needs to be more thoroughly addressed (Benine et al. 2009).

Chromosome studies in the genus *Moenkhausia* are still restricted and cytogenetic data are available only for six species (Portela-Castro et al. 2001). In *Moenkhausia sanctaefilomenae*, a stable diploid number of 50 chromosomes and few karyotype variations among the different populations analyzed have been reported. Furthermore, some populations of *Moenkhausia sanctaefilomenae* can show a high inter- and intra-individual variability of the NOR (nucleolus organizer region) phenotypes, as well as conspicuous blocks of constitutive heterochromatin in the pericentromeric region of the chromosomes (Foresti et al. 1989, Portela-Castro et al. 2001, Portela-Castro and Júlio Jr. 2002). However, the occurrence of several B microchromosomes in the genome of this species is the most peculiar feature to be studied in this fish group (Foresti et al. 1989).

B chromosome includes a variety of extra chromosomes that display conspicuous heterogeneity in their nature, behavior, and evolutionary dynamics. This definition highlights some of the most universal properties of B chromosomes: their dispensability (that is, they are not necessary for the host to complete a normal life cycle); their origin from chromosomes (either from within the same species or from other species); and their remarkable differentiation relative to A chromosomes, with which they do not recombine (Camacho 2005).

B chromosomes are widely distributed among eukaryotes and their occurrence has been reported in 10 species of the fungi, nearly 1.300 plants (more than 1.400 when different ploidy levels of the same species are considered separately), and over 500 animals (Camacho 2005). In addition, B chromosomes have been described in 61 species of Neotropical fish to date (Carvalho et al. 2008).

In species of *Moenkhausia*, B chromosomes were documented for *Moenkhausia sanctaeﬁlomenae* and *Moenkhausia intermedia* Eigenmann, 1908 (Portela et al. 1988, Foresti et al. 1989). Differently from other microchromosome-bearing fish species, which exhibit a low frequency and a sporadic occurrence (Hashimoto et al. 2008, Oliveira et al. 2009, Hashimoto et al. 2011), several microchromosomes can be found in the genome of *Moenkhausia sanctaeﬁlomenae* and, in certain situations, the frequency can be related to sex (Portela-Castro et al. 2001).In fact, in Neotropical fish, it is possible to find both B macrochromosomes and B microchromosomes (Oliveira et al. 2009), but in both cases the presence of a large number of B chromosomes in the cells is rare, as was observed in *Prochilodus lineatus* (Valenciennes, 1836)and *Moenkhausia sanctaeﬁlomenae*, which presented up to eight microchromosomes in the cells (Foresti et al. 1989, Voltolin et al. 2011).

Another interesting characteristic observed in the B microchromosomes of *Moenkhausia sanctaeﬁlomenae* is the polymorphism revealed by C-banding. Through this method, these microchromosomes can be characterized in different classes according to the pattern of constitutive heterochromatin; they can be partially and totally heterochromatic, and euchromatic (Foresti et al. 1989). Thus, such polymorphism indicates a distinct DNA composition between these microchromosomes, especially of repetitive DNA.

In the present study, we carried out cytogenetic analyses in one particular population of the fish *Moenkhausia sanctaeﬁlomenae* focusing on two special features concerning the B microchromosomes: the occurrence of nucleolar activity in the B chromosome of this species and a study about the maintenance of microchromosomes in this population through the calculation of the mitotic instability index (MI).

## Material and methods

The cytogenetic analyses were carried out in chromosomal preparations obtained from 15 specimens (8 males and 7 females) of *Moenkhausia sanctaefilomenae*. The individuals were collected from a population of the Batalha River (22°7.02'S, 49°16.01'W), belonging to Tietê River basin, São Paulo State, southeastern Brazil. The voucher specimens were identified and stored in the fish collection of the Laboratório de Genética de Peixes, UNESP, Bauru, SP, Brazil.

Before sacrifice, the animals were inoculated with yeast cell suspension to increase the number of metaphase cells (Oliveira et al. 1988). Chromosomal preparations were obtained from gill and kidney tissues using the technique described by Foresti et al. (1993). Silver staining (Ag-staining) of the nucleolus organizer regions followed the technique of Howell and Black (1980), and C-banding was performed according to Sumner (1972). The chromosomal morphology was determined on the basis of arm ratio, as proposed by Levan et al. (1964) and the chromosomes were classified as metacentric (m), submetacentric (sm), subtelocentric (st), and acrocentric (a).

The index to quantify the mitotic instability of B chromosome, MI, which was calculated as the sum of the absolute values of every deviation in B number with respect to the median (M), and normalized by dividing the median and the number of cells analyzed (N) so that the index is independent of the number of B and the sample size were performed by means of one-way ANOVA.

**MI = (M-ni/fi)/M.N**

where ni is the numer of B chromosome in the different types of cells that do not coincide with M, and fi is the number of cells of each particular type.

## Results and discussion

**Figure 1.**
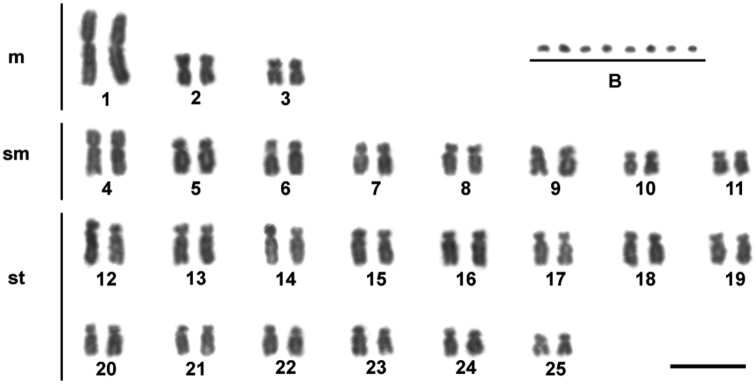
Giemsa-stained karyotype showing 2n = 50 chromosomes of one individual of *Moenkhausia sanctaefilomenae*. In evidence, eight B microchromosomes. Bars = 10 µm.

In the individuals of *Moenkhausia sanctaefilomenae*, our results showed a diploid chromosome number of 50 chromosomes, with karyotypes composed of 6 m, 16 sm and 28 st (fundamental number FN = 100) (Fig. 1). No sex-related karyotype difference was observed. The diploid chromosome number and the karyotypes composed mainly of metacentric and submetacentric chromosomes seem to be a conserved characteristic observed for different *Moenkhausia sanctaefilomenae* populations (Foresti et al. 1989, Portela-Castro et al. 2001, Portela-Castro and Júlio Jr. 2002).

Extra chromosomes were observed in the genomes of all individuals of *Moenkhausia sanctaefilomenae*, which were characterized as B microchromosomes (Fig. 1). We detected inter- and intra-individual variation in relation to the number of B chromosomes in the cells, with specimens bearing up to eight microchromosomes. Metaphase counts for 13 individuals showing the variation in supernumerary chromosome numbers are presented in Table 1. The modal numbers were of 2 and 3 microchromosomes. Such variation is in accordance with the pioneer study of Foresti et al. (1989), who also analyzed a population from the Tietê River basin. On the other hand, the specimens from the Paraná River analyzed by Portela-Castro et al. (2001), showed differences because the presence of 0–2 microchromosomes were reported only in males. These polymorphisms concerning the distribution of B chromosomes indicate a process of genetic divergence in distinct populations that likely occurs in some species restricted to small tributaries and streams, as reported for species of *Astyanax* (Moreira-Filho and Bertollo 1991, Vicari et al. 2008, Hashimoto et al. 2011).

**Table 1.** Metaphase counts for 13 specimens of *Moenkhausia sanctaefilomenae* demonstrating the variation in B microchromosome numbers.<br/>

**Specimen identification**	**Number of B microchromosomes per cell**									**Number of cells counted**
	**0**	**1**	**2**	**3**	**4**	**5**	**6**	**7**	**8**	
849	6	12	22	-	-	-	-	-	-	40
852	2	32	36	10	-	-	-	-	-	80
853	9	70	-	-	-	-	-	-	-	79
857	3	3	6	10	9	9	2	-	-	42
887	-	3	12	22	4	2	7	13	2	65
888	-	6	6	41	5	10	-	-	-	68
889	-	3	12	63	11	3	-	-	-	92
1233	-	4	10	24	26	29	13	4	-	110
1235	1	6	31	79	15	8	-	-	-	140
1240	8	31	33	26	3	-	-	-	-	101
1241	9	137	175	4	-	-	-	-	-	325
1242	24	85	27	-	-	-	-	-	-	136
1246	5	25	4	5	4	1	-	-	-	44

**Figure 2.**
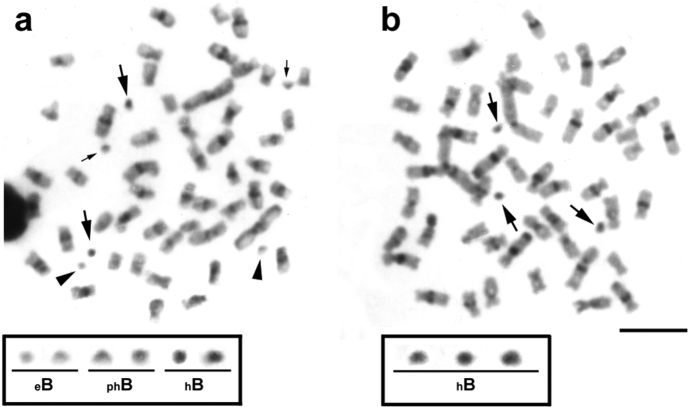
Metaphases from specimens of *Moenkhausia sanctaefilomenae* after C-banding technique. In (**a**), metaphase shows euchromatic (_e_B), partially heterochromatic (_ph_B) and totally heterochromatic (_h_B) microchromosomes. In (**b**), metaphase demonstrates only heterochromatic B chromosomes. The boxes show enlarged B chromosomes. Major and minor arrows indicate totally and partially heterochromatic B microchromosomes, respectively. Arrowheads exhibit euchromatic B microchromosomes. Bars = 10 µm.

Analysis of the constitutive heterochromatin patterns by C-banding showed heterochromatic blocks in the centromeric and pericentromeric regions in the majority of the chromosomes (Fig. 2a, b). Such general heterochromatin pattern was also observed in previous analyses for other *Moenkhausia sanctaefilomenae* populations (Foresti et al. 1989, Portela-Castro et al. 2001, Portela-Castro and Júlio Jr. 2002), demonstrating that these chromosomal regions present a highlyconservative distribution in this species. The supernumerary chromosomes showed different C-banding patterns. We observed euchromatic (Fig. 2a) as well as partially or totally heterochromatic microchromosomes (Fig. 2a, b), evidencing that these B chromosomes can have a different DNA composition, mainly of repetitive sequences. This is a common feature also reported for B chromosomes in other characid species (Néo et al. 2000, Jesus et al. 2003, Moreira-Filho et al. 2004).

**Figure 3.**
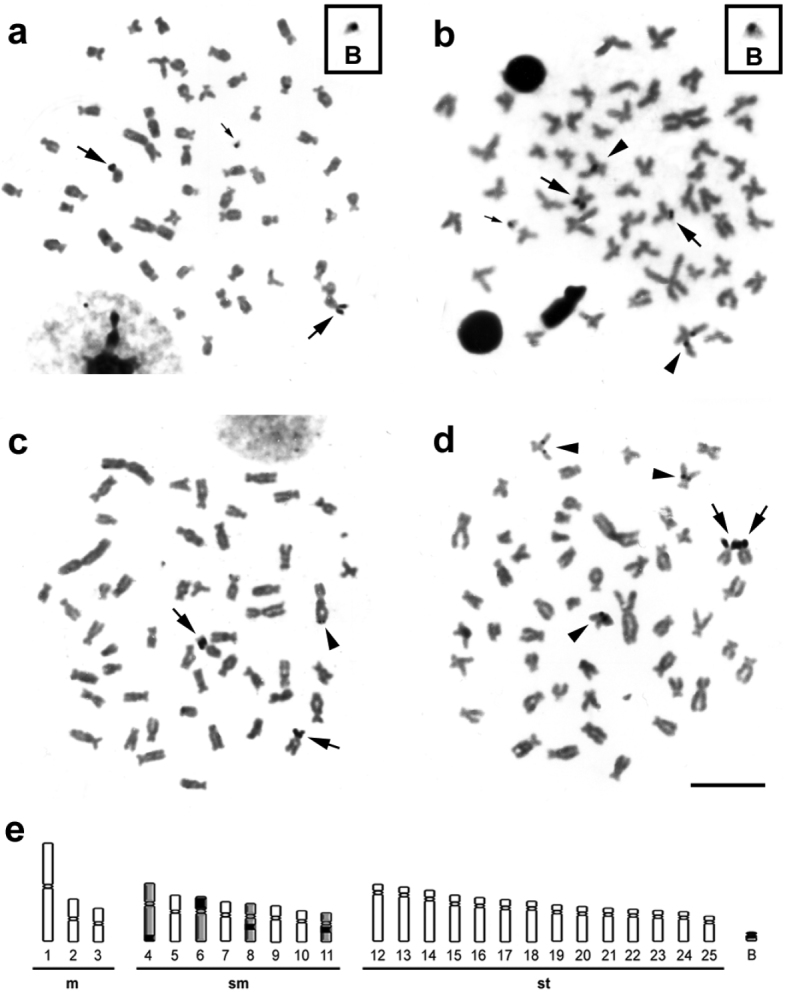
Metaphases from specimens of *Moenkhausia sanctaefilomenae* submitted to the silver coloration. In (**a**) and (**b**), metaphases of one individual show intra-individual variability of active NORs. The boxes exhibit enlarged B chromosomes with nucleolar activity. In (**c**) and (**d**), metaphases of different samples demonstrate inter-individual variability for the NORs. In (**e**), schematic representation shows the NOR-bearing chromosomes (4, 6, 8, 11 and B). Major arrows indicate major NOR-bearing chromosomes (chromosomes 6). Minor arrows show nucleolar activity in the B microchromosomes (**a**) and (**b**). Arrowheads exhibit minor NORs demonstrating a variable pattern of activity in different chromosomes. Bars = 10 µm.

The Ag-impregnation revealed intra- (Fig. 3a, b) and inter-individual (Fig. 3c, d) variability for the NOR phenotypes in metaphases of *Moenkhausia sanctaefilomenae*, ranging from two to five Ag-positive sites, distributed in the interstitial and terminal regions of distinct chromosomes (Fig. 3e). However, the chromosomes 6 always presented active Ag-NORs, and consequently, were considered the major NOR-bearing chromosomes. The minor NORs showed a very variable pattern of activity. Such NOR features were previously reported by Foresti et al. (1989).

Indeed, NOR expression was detected in a B chromosome of one individual, which carried only this microchromosome (Fig. 3a, b). We analyzed 60 cells by Ag-staining and observed that about 20% had active ribosomal DNA sites in the B chromosome of this individual. Moreover, this supernumerary chromosome showed to be euchromatic by C-banding. The nucleolar region is a dynamic cell compartment involved in the control of numerous cellular functions that can be visualized after Ag coloration, when the genes present activities in the interphase that anticipates the mitosis (Roussel et al. 1996, Caperta et al. 2007, Hiscox 2007). Therefore, Ag-staining provides a simple and reliable method to detect ribosomal RNA (rRNA) gene transcription (Bakkali et al. 2001, Teruel et al. 2009). B chromosomes in several species carry rRNA genes (Camacho 2005), including fish species (Baroni et al. 2009, Poletto et al. 2010), and in most of the cases, rRNA has been detected by Ag-staining evidencing the presence of active genes, as demonstrated in the present study. However, further analysis using FISH technique will be necessary to detect positions of additional rDNA genes not only during their activity.

The fact that the NORs located in the chromosomes 6 were always active can suggest that a process of nucleolar dominance can influence the rRNA gene transcription in order to provide the proper amount of rRNA for ribosome assembly. Nucleolar dominance is an epigenetic phenomenon common in interspecific hybrids, in which ribosomal RNA genes set inherited from one parental are rather transcribed in relation to the other (Hashimoto et al. 2009). Nucleolar dominance can also be a consequence of the regulatory process that controls the effective dosage of rRNA genes in pure species (non-hybrid) (Pikaard 2000). Nowadays, the mechanisms by which whole NORs or rRNA genes subsets are selected for inactivation still remains unclear (Preuss and Pikaard 2007).

The chromosome context appears to be important for NOR activity, as deduced from changes in the on/off activity status following chromosome rearrangements moving NORs to new locations (Pikaard 2000). The present findings show that the B chromosome plays an important role in the genome organization of *Moenkhausia sanctaefilomenae*, and will be useful for further analyses to determine whether the frequency of B chromosomes expressing their NOR is changing over time and how the B chromosome context can influence A chromosome NOR activity.

In relation to the mitotic instability and maintenance of B chromosomes in *Moenkhausia sanctaefilomenae*, we compared the results reported by Foresti et al. (1989) with the data described in this study, because both populations were collected from the Tietê River basin (Brazil). In both populations, a pattern of mitotic instability for all analyzed individuals was observed. The analysis of the standard maintenance of these B chromosomes by calculating the mitotic instability index (MI) revealed that the *Moenkhausia sanctaefilomenae* population analyzed by Foresti et al. (1989) showed a MI = 0.6; however, the *Moenkhausia sanctaefilomenae* population analyzed in the present study showed a MI = 0.2. Taking account the high variability of B chromosomes in the genomes of these *Moenkhausia sanctaefilomenae* specimens, further studies are still necessary to verify if these B chromosomes might be underway towards the neutralization stage, in accordance with the life cycle of B chromosomes described by Camacho et al. (1997).

In fish, the possibility of neutralization through mitotic stabilization of B-chromosomes was also observed in *Prochilodus lineatus*, in the population from the Mogi-Guaçu River (Brazil) (Oliveira et al. 1997). Afterwards, in this same population, Cavallaro et al. (2000) found a drastic temporal decline in the degree of B mitotic instability; Voltolin et al. (2010) showed that the stabilization process was continuous for over 15 years; and currently, the population of *Prochilodus lineatus* from the Mogi-Guaçu River presents a total mitotic stability index (MI = 0) and the B chromosomes were considered completely neutralized.

In Neotropical fish, most of the studies about B chromosomes are still descriptive, because many species have not yet been cytogenetically analyzed. Thus, studies focusing B chromosomes in Neotropical fish are extremely necessary to better understand this intriguing class of chromosomes, as has been done for some species, such as *Prochilodus lineatus* and *Astyanax* species (Moreira-Filho et al. 2004, Voltolin et al. 2010, 2011, Hashimoto et al. 2011), towards which efforts are more thoroughly addressed. Thus, our results show that B chromosomes of *Moenkhausia sanctaefilomenae* are excellent models and also that extensive studies in this species are essential to improve the knowledge of the diversification of B chromosomes.
